# Tracking orthographic learning in children with different profiles of reading difficulty

**DOI:** 10.3389/fnhum.2014.00468

**Published:** 2014-07-04

**Authors:** Hua-Chen Wang, Eva Marinus, Lyndsey Nickels, Anne Castles

**Affiliations:** Department of Cognitive Science, ARC Centre of Excellence in Cognition and its Disorders, Macquarie UniversitySydney, NSW, Australia

**Keywords:** orthographic learning, developmental dyslexia, subtypes, phonological decoding, orthographic knowledge

## Abstract

Previous studies have found that children with reading difficulties need more exposures to acquire the representations needed to support fluent reading than typically developing readers (e.g., Ehri and Saltmarsh, 1995). Building on existing orthographic learning paradigms, we report on an investigation of orthographic learning in poor readers using a new learning task tracking both the accuracy (untimed exposure duration) and fluency (200 ms exposure duration) of learning novel words over trials. In study 1, we used the paradigm to examine orthographic learning in children with specific poor reader profiles (nine with a surface profile, nine a phonological profile) and nine age-matched controls. Both profiles showed improvement over the learning cycles, but the children with surface profile showed impaired orthographic learning in spelling and orthographic choice tasks. Study 2 explored predictors of orthographic learning in a group of 91 poor readers using the same outcome measures as in Study 1. Consistent with earlier findings in typically developing readers, phonological decoding skill predicted orthographic learning. Moreover, orthographic knowledge significantly predicted orthographic learning over and beyond phonological decoding. The two studies provide insights into how poor readers learn novel words, and how their learning process may be compromised by less proficient orthographic and/or phonological skills.

## Introduction

Orthographic learning has been defined as the transition from the slow sounding out of an unfamiliar new word to the rapid automatic recognition of the same word. It is widely acknowledged that beginning readers need to make this transition in order to become proficient readers (e.g., Ehri and Wilce, 1983; Share, 1995; Castles and Nation, 2008). In this study, we explored orthographic learning in children with poor reading ability and investigated the factors that are associated with their success in acquiring new orthographic representations.

Most developmental theories propose that the sounding out of words, phonological decoding, is an important mechanism for reaching the final stage of automatic reading (for a review, see Ehri, 2005). Among these theories, the self-teaching hypothesis is associated with a strong claim for the importance of phonological decoding in orthographic learning (Share, 1995, 1999). It proposes that phonological decoding is the first and most important step of orthographic learning, providing an opportunity for this learning to take place. The act of phonological decoding is proposed to allow the reader access to a word's spoken form, as well as to draw their attention to the order and identity of the letters. This, together with repeated exposure to the new word, assists the reader in establishing an orthographic representation. According to the self-teaching hypothesis, although phonological decoding is crucial in orthographic learning, it is not the only factor: there is a secondary, orthographic processing component, which also determines the success of orthographic learning, although the nature of this mechanism is little understood (Share, 1995, 2011).

If phonological decoding is important for acquiring orthographic representations, proficient phonological decoding processes should increase the likelihood of successful orthographic learning. Conversely, impaired phonological decoding processes should be expected to lead to difficulties in orthographic learning. Indeed, abundant studies seem to support the view that deficits in phonological processing skills may be a primary cause of reading difficulties (e.g., Rack et al., 1992; Stanovich and Siegel, 1994) as well as orthographic learning difficulties (Share and Shalev, 2004).

However, a large body of evidence on heterogeneity within the dyslexic population and on the existence of different subtypes of developmental dyslexia suggests that the relationship between phonological decoding skills and orthographic learning may not be straightforward (Castles and Coltheart, 1993; Manis et al., 1996; Stanovich et al., 1997; Valdois et al., 2003; Castles et al., 2010b; Jones et al., 2011; McArthur et al., 2013; Peterson et al., 2013). The outcomes of these studies show that impairment in phonological decoding and impairment in automatic whole-word recognition can occur selectively—one aspect of reading can be impaired while the other develops normally. Namely, children with a *surface dyslexia* profile struggle to read irregular words (e.g., *yacht*) but are not impaired in reading nonwords (e.g., *grep*). This indicates that they are specifically impaired in recognizing whole words, whilst their phonological decoding skills are intact. Conversely, children with a *phonological dyslexia* profile show impairments in nonword reading but not irregular word reading, indicating a specific impairment in phonological decoding processes while being intact in sight word recognition skills. Note that children with these profiles are seen as falling at the ends of a normal continuum of ability on the relevant reading subskills, not as qualitatively distinct subtypes.

In sum, based on the view that phonological decoding is the primary factor in successful orthographic learning, the reading profiles of surface dyslexia and phonological dyslexia seem to be somewhat of a paradox. How are some beginning readers able to build up orthographic knowledge despite poor decoding skills (phonological dyslexics) and why do some children with proficient decoding skills fail to build up orthographic representations (surface dyslexics)? In order to address these questions we need to examine how orthographic learning occurs in these two subtype profiles.

To date, only two studies have explicitly contrasted differences in orthographic learning in children with phonological and surface dyslexia. Castles and Holmes (1996) found that, as expected by their specific reading profiles, children with a surface dyslexia profile were poorer at learning novel irregular words (measured by an orthographic choice task in which the child had to choose the target item from its distracters) than children with a phonological dyslexia profile. However, since the novel words in their study were all irregular, it may be that children with a surface profile performed more poorly than children with a phonological profile because their usual phonological decoding strategy is not effective for such items (e.g., how would one phonologically decode a word like “laugh”?).

Bailey et al. (2004) built on the results of Castles and Holmes (1996) by comparing orthographic learning of both regular and irregular words in children with profiles of phonological dyslexia and surface dyslexia. They found that overall, the two profiles were no different from each other, but were both more impaired than chronological age controls in orthographic learning (as measured by reading accuracy). In addition, they found that both children with a surface profile and the controls showed an advantage in learning regular words as compared to irregular words. In contrast, children with a phonological profile showed no difference between regular and irregular words, suggesting that phonological decoding was not relied on during orthographic learning. Although this study provided more insight into the orthographic learning processes of these two profiles of dyslexia, there are still some limitations. First, orthographic learning results were based on reading accuracy only^1^. Thus, the finding that children with a surface profile were more accurate in reading regular than irregular words may have been a function of the “decodability” of the words rather than orthographic learning *per se* (e.g., *cat* can be read correctly by phonological decoding, whereas this is not possible for *yacht*). Hence, to determine whether children with surface dyslexia are indeed better at acquiring (and not just decoding) regular orthographic representations than irregular ones, improved measures of orthographic learning with minimal influence from decoding ability are required. Second, selection of the subgroups was based on a relatively lax criterion. Instead of selecting children with a surface profile that were impaired on irregular word reading only, and phonological profile children that were impaired on nonword reading only, Bailey and colleagues based their selection on a discrepancy score between nonword and irregular word reading. Hence, for example, a phonological profile child in their study could have been poor on both nonword and irregular word reading, but with irregular word being relatively better than nonword reading. The design of the study presented here allows us to address these problems.

The first aim of the present study was to further extend our understanding of orthographic learning in children with surface and phonological profiles. By studying orthographic learning in these two subgroups we also aimed to bridge the gap between previous work on orthographic learning (mostly conducted with normal readers) and the extensive literature on subtypes in dyslexia. Building on the studies of Castles and Holmes (1996) and Bailey et al. (2004), we used a more stringent subgroup criterion in selecting participants and developed a novel paradigm to explore orthographic learning. Just like Bailey et al. we included a sample of typical readers as controls so that we could not only compare the performance of the children with different profiles, but also contrast their performance to that of normal readers.

We also included a broader range of measures of orthographic learning than in the previous studies. Given that spelling tasks are often difficult for poor readers, we included an orthographic choice task. Finally, we developed a new learning paradigm that assesses reading accuracy under both untimed and time-limited exposure conditions. Time-limited exposure reading accuracy is interpreted here as a fluency measure of item specific orthographic knowledge, as rapid recognition of words is considered a hallmark of the acquisition of orthographic representations (Yap and van der Leij, 1993; Marinus et al., 2012). This reasoning is similar to the idea that the time that is required to read a word is reduced when a word is read as a whole unit rather than by phonological decoding (e.g., Coltheart, 1983; Ehri, 2005). An additional benefit of this paradigm is that it allowed us to tap orthographic learning by tracking improvement of fluency over learning cycles. Hence, we were able to monitor orthographic learning in a dynamic and ongoing fashion. Finally, just like Bailey and colleagues, we included both regular and irregular words in order to see if we could replicate the regularity effect for children with a surface profile, and the absence of a regularity effect for children with a phonological profile.

In our novel word-learning paradigm, novel letter strings were assigned regular or irregular pronunciations and presented in three learning cycles. After each cycle, we measured reading accuracy under both untimed and time-limited stimulus exposure duration. After the three cycles were completed, traditional spelling and orthographic choice tasks were administered. The untimed reading condition provided the opportunity for children to decode and build up orthographic representations of the novel words. In order to measure whether orthographic learning had taken place, each untimed reading block was followed by a time-limited exposure block in which items were presented for only 200 ms.

As mentioned earlier, this paradigm not only allows us to explore whether the two groups with contrasting reading profiles differ in their orthographic learning performance, but also to examine whether and to what extent orthographic learning improves with number of learning exposures. Previous studies examining the transition from decoding to rapid word recognition (as measured by increases in reading speed) have found that children with dyslexia need many more exposures to acquire novel word representations than typically developing readers (Reitsma, 1983; Manis, 1985; Ehri and Saltmarsh, 1995). Reitsma (1983, Experiment 3) reported that even six exposures to novel words was not enough to result in any increase in reading speed (taken as an index of orthographic learning) in a group of children with dyslexia. In contrast, in the same experiment, a group of younger readers without reading difficulties showed a steep increase in word reading speed. Note that none of these studies made a distinction between different profiles of reading difficulty. Hence, we used the current paradigm to monitor orthographic learning within two groups of poor readers with contrasting reading profiles.

Using the same paradigm, but with a larger sample of poor readers, we conducted a second study to explore to what extent different reading and language skills predict orthographic learning. For this purpose, we drew on an explicit model of component processes involved in skilled reading, the dual-route model of reading aloud (Coltheart et al., 1993, 2001). The six components of this model include: letter analysis; letter-sound conversion, phonemic buffer, orthographic lexicon, semantics, phonological lexicon. We used regression analyses to investigate the association between these components and orthographic learning. Reading and language skills mapping onto the six different components were used as predictors, and the orthographic learning results were used as outcome measures.

## Study 1

As outlined in the Introduction, the existence of children with surface and phonological reading profiles challenges the role of phonological decoding in orthographic learning. The aim of Study 1 was to investigate how orthographic learning takes place in poor readers with contrasting reading profiles. Study 1 consisted of two parts. The first part aimed to validate the group membership of the phonological and surface profiles. In order to do this we measured language and reading skills involved in reading processes based on the dual-route model of reading aloud. In the context of the dual route model, we expect the children with a phonological profile to be impaired in the letter-sound knowledge process of the nonlexical route. In contrast, children with a surface profile are thought to be impaired in the lexical route, the orthographic lexicon in particular.

In the second part of Study 1, we used the novel word learning paradigm to investigate orthographic learning of the two profiles. The questions of interest were: (1) Are these children able to learn novel words at all? If so, is their learning rate slower than controls? This part of the study aimed to replicate previous studies suggesting that children with dyslexia are impaired at orthographic learning (Reitsma, 1983, 1989) (2). Will children with a phonological profile, having impaired phonological decoding skills, be less efficient at learning novel words than control children and those with a surface profile? Alternatively, will children with a phonological profile learn novel words faster than children with a surface profile as predicted by their subtype reading profiles? (3) Will children with phonological and surface profiles differ in the size of the regularity effect? Typically developing children have been shown to learn regular words better than irregular words as regular words are more “phonologically decodable” than irregular words (Wang et al., 2012). However, as suggested by Bailey et al. (2004), children with a phonological profile may show no effect of regularity on orthographic learning due to their impaired phonological decoding skill. Instead, they may learn novel words via some kind of rote association between the sound and the form of the novel words bypassing the phonological decoding process. Children with a surface profile, in contrast, may show a normal word regularity effect on orthographic learning as they have average phonological decoding skills.

### Participants

Ninety-one poor readers (average age 9.3, range 7.2–12.3) were recruited from schools, clinics or via newspaper advertisements to participate in a reading training study at Macquarie University. Children were included in the study if they scored at least one standard deviation below average for their age on one or both subscales (irregular word and nonword reading) of the Castles and Coltheart 2 test (CC2; Castles et al., 2010a). All poor readers scored within the normal range on non-verbal IQ (Kaufman Brief Intelligence Test, K-Bit; Kaufman and Kaufman, 1990).

From this larger sample we selected two groups of poor readers: one with a surface profile and one with a phonological profile. We will from here refer to them as the “surface group” and the “phonological group.” The criteria for a surface profile were performance within the normal range (z-score > −1.00) on nonword reading accuracy and below average performance on irregular word reading (z-score < −1.00, which is equivalent to the bottom 15% of the norms). In addition, to ensure a discrepancy in skills, the z-score difference between nonword and irregular word reading had to be more than 0.5. The same test was administered twice in two sessions that were 8 weeks apart. Only children with consistent reading profiles across the two sessions were included. Nine poor readers fitted our stringent criteria of a surface profile on both testing sessions. Next, we identified children showing consistent profiles of phonological dyslexia (nonword z-score < −1.00, irregular word > −1.00, with a difference of more than 0.5), resulting in a subsample of 22. From this sample we selected nine participants with a phonological profile, matching the surface group in age, IQ and level of impairment on the relevant reading subtest. Finally, we recruited nine age-matched typical readers that were participating in reading studies at Macquarie University as controls. The reading accuracy of these controls was within one standard deviation below the average range and 1.5 standard deviation above the average range scores on all three subscales of the CC2 (please see Table 1 for the characteristics of the three groups).

**Table 1:** **Characteristics and reading processing skills of the control, phonological, and surface groups**.

	**Controls**	**Phonological profile**	**Surface profile**	**Phon. vs. Surf**.	
	**Mean (*SD*)**	**Mean (*SD*)**	**Mean (*SD*)**	***t***	***p* (2-tailed)**
**CHARACTERISTICS**					
Age	9.31 (1.52)	9.49 (1.35)	9.42 (1.62)	0.11	0.92
Nonverbal IQ (K-Bit, standardized score/100)	*NA*	105.22 (8.60)	101.44 (13.48)	0.71	0.49
Nonword reading (CC2, z-score)	0.15 (0.66)	−1.58 (0.25)^**^	−0.51 (0.44)^*^	−6.31	0.00^**^
Irregular word reading (CC2, z-score)	0.29 (0.55)	−0.57 (0.21)^**^	−1.53 (0.24)^**^	8.94	0.00^**^
**READING PROCESSING SKILLS**					
Letter analysis (Cross-case matching, raw score/14)	13.57 (1.13)	13.78 (0.44)	13.00 (1.58)	0.34	0.73
Letter-sound knowledge (LeST, raw score/51)	41.67 (3.61)	34.11 (7.22)^*^	40.78 (4.06)	2.42	0.03^*^
Orthographic lexicon (DOOR/DOAR, raw score/30)	22.89 (4.89)	24.44 (2.01)	18.89 (3.72)^+^	3.94	0.00^**^
Semantics (PPVT, standardized score/100)	104.57 (13.97)	96.56 (7.99)	96.56 (12.82)	0.00	1.00
Phonological Lexicon (ACE, standardized score/10)	8.86 (2.04)	7.89 (1.54)	7.67 (2.96)	0.20	0.85
Phonemic Buffer (NEPSY, standardized score/10)	9.43 (3.65)	8.22 (2.22)	10.11 (1.45)	2.13	0.05^+^

*The asterisks in the “phonological profile” and “surface profile” columns indicate significant differences compared to control group*.

* *p < 0.05*,

** *p < 0.01*,

+ *p < 0.1*.

### Subgroup validation

**Figure 1:**
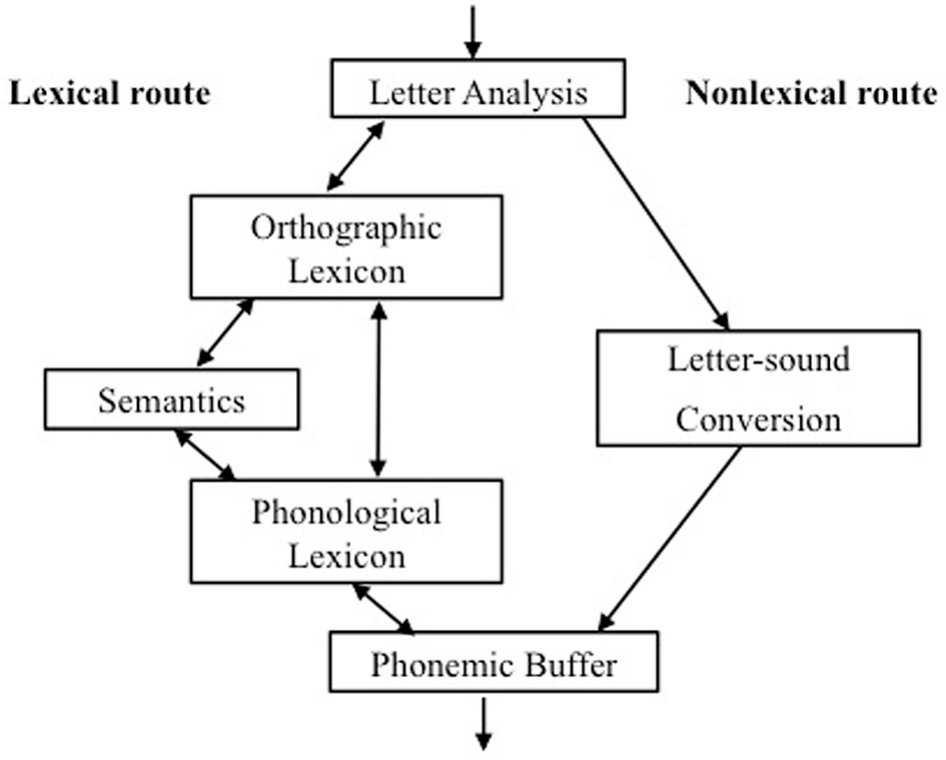
**The dual route model and its six basic components**.

In this first part of Study 1, we validated subgroup membership by examining the language and reading skills of the two groups with contrasting reading profiles. We designed tasks that aimed to tap different components of the dual route model of reading aloud (see Figure 1). This model proposes a *lexical* route through which words are directly recognized as whole units and a *nonlexical* route through which words are decoded phonologically.

As can be seen from Figure 1, each of these routes consists of a number of processing components, some shared across the routes and some separate. When a reader sees a printed word, the letters will first be recognized in the *letter analysis* component. Then in the nonlexical route, the graphemes of the word are phonologically decoded by the *letter-sound knowledge* component (also referred to as the “grapheme-to-phoneme conversion” component). In the lexical route, the orthography of known words is activated as a whole unit in the *orthographic lexicon*. Subsequently, in the *semantic system*, the meaning of the word is activated and then in the *phonological lexicon* the sound of the word is activated. The final component of the model is the *phonemic buffer* where phonemes are activated and temporarily stored before they are spoken.

#### Measures of reading processes

Each of the six basic components in the dual-route model was assessed with one test as described in the sections below. Test–retest reliability (Pearson's *r*) is reported for each measure based on scores over two testing sessions that are 8 weeks apart, with a sample of 115 children, aged 7–12 in a larger reading training study (McArthur et al., 2013).

#### Letter analysis

Letter analysis was measured with a cross-case copying task (McArthur et al., 2013). This task consists of 14 letters, 7 in upper case and 7 in lower case. For lower case letters the child was asked to write down the upper case of the same letters (e.g., t − T), and vice versa for upper case letters. Test–retest reliability, *r* = 0.75.

#### Letter-sound knowledge

The ability to convert letters or letter strings into sounds was tested with the Letter-Sound Test (LeST, Larsen et al., 2011). Each child was asked to produce the appropriate sound for 51 single-letter and multiletter graphemes. The items were presented on individual flash cards. The graphemes were selected as being consistent, in other words they had the same pronunciation in more than 75% of occurrences of that grapheme according to the CELEX database (Baayen et al., 1993). Test–retest reliability, *r* = 0.84.

#### Orthographic lexicon

Word-specific orthographic knowledge was assessed with the DOOR/DOAR lexical decision test (McArthur et al., 2013). Thirty target words, ranging in frequency from 3 to 625 instances per million words, were selected from the Children's Printed Word Database (CPWD, Masterson et al., 2003). All words were selected to have alternative, homophonic spellings with adjustments of the vowel (e.g., FLAME changed to FLAIM) or a consonant (e.g., CURL changed to KURL). Each item was presented paired with its alternative homophonic spelling (e.g., DOOR and DOAR). The child was asked to circle the correct spelling. Test–retest reliability, *r* = 0.57.

#### Semantics

Semantic knowledge was measured with the Peabody Picture Vocabulary Test 4 (PPVT-IV, Dunn and Dunn, 2007). For each item the child was presented with four pictures and asked to point to the picture that was named by the tester. The administration of the test was stopped when the child made more than eight errors in a set of 12 items. Scores were standard scores with a mean of 100 and a standard deviation of 15. Test–retest reliability, *r* = 0.84.

#### Phonological lexicon

The ability to access the phonological lexicon was measured with the Naming subtest of the Assessment of Comprehension and Expression (ACE6–11 test; Adams et al., 2001). The child was asked to name 25 pictures. No stopping rule was applied. Test–retest reliability, *r* = 0.87.

#### Phonemic buffer

We tested the phonological output buffer with a standardized nonword repetition task, a subtest of the NEuroPSYchology (NEPSY) test (Korkman et al., 1998). In this task, the child was asked to listen to and orally repeat digitally recorded nonwords (e.g., crumsee). Scores were standard scores with a mean of 10 and a standard deviation of 3. Test–retest reliability, *r* = 0.72.

### Results: subgroup validation

Table 1 presented the performance of the surface, phonological and control groups on the selection measures and the other measures of reading processing skills. The two groups were significantly different on the selection measures: nonword and irregular word reading accuracy. In addition, and as would be predicted, the phonological group performed significantly more poorly on the letter-sound knowledge test and the surface group performed significantly more poorly on the orthographic knowledge test (DOOR/DOAR). In addition, the difference on the nonword repetition test (NEPSY) approached significance (*p* = 0.05), with the surface group appearing to outperform the phonological group. However, as both groups still performed within the normal range on this task, this result is not discussed further. The two groups did not differ on any other measure. The results of the assessment of reading processing skills therefore confirmed that the phonological group had inferior letter-sound knowledge in the nonlexical route and the surface group showed lower proficiency of the orthographic knowledge in the lexical route.

### Orthographic learning task

#### Materials

This task consisted of eight four to five-letter nonwords (e.g., *vack*), four of which were assigned with regular pronunciations and the other four with irregular pronunciations (please see Supplementary Material). The nonwords were created in the same way as the items used in Wang et al. (2011), but the items are not identical to Wang et al. due to differences in the experimental design. The regular items were pronounced according to a set of typical grapheme-phoneme correspondence rules (Rastle and Coltheart, 1999). “Typical” was defined on the basis that the pronunciation of the vowel occurred in more than 50% of words containing that vowel grapheme in both the CELEX database (Baayen et al., 1993) and the CPWD (Masterson et al., 2003). The irregular nonwords had pronunciations that did not follow typical letter-sound rules: the allocated pronunciation of the vowel in the target word occurred in fewer than 50% of words in the CELEX and the CPWD. All of the irregular pronunciations were nevertheless existing grapheme-phoneme correspondences in English. However, the pronunciations were infrequent and did not occur in the context of the vowels and the final consonants (bodies) of the irregular nonwords that were used in this task. For example, the nonword *cleap* was assigned a pronunciation “claip”; *ea* is pronounced this way in, for example, *great, break*, but is always pronounced “ee” when followed by *–p* (e.g., *heap, leap*).

#### Procedure

**Figure 2:**
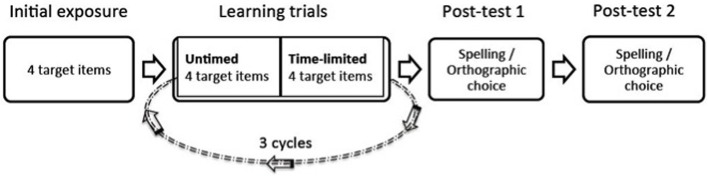
**Procedure of the orthographic learning task**.

Children were tested individually in a quiet room. They learned four regular items followed by four irregular items. For both regular and irregular words, the same procedure was used an initial exposure phase, learning trials and two post-tests (see Figure 2).

During the initial exposure phase, the child was first presented with a picture with elves and was told they were going to learn the names of some of these elves. Next, the tester introduced the spoken forms of the four target nonwords (“elves names”) to the children (initial exposure). This was necessary in order to expose the children to the pronunciations of the irregular nonwords. After this, the child was seated in front of a computer and the nonwords appeared on the screen one at a time. During the first presentation on the computer screen the tester said: “The name of this elf is ____.” The children were not asked to read or repeat the novel words at this point and no accuracy was recorded. After a nonword had been introduced to the child orally and in print in the exposure phase, the first cycle of the learning trials began and reading accuracy was recorded. The four nonwords would appear on the screen one by one in a randomized order, and the child was asked to read them aloud. This was the untimed exposure reading. Feedback was provided regardless of whether the child read the target word correctly or not, to give an equal number of phonological exposures to each word. For example, after each response was given by the child, the experimenter said, “that's correct, it's a ferb” for correct responses; or “not quite, it is a ferb” for incorrect responses.

All three rounds of untimed reading were followed by a block of time-limited reading (200 ms presentation, with #### as backward masks) of the target words. This set up allows us to obtain an ongoing measure of orthographic learning (i.e., the ability to recognize words instantaneously) after each exposure (i.e., untimed reading with plenty of time to decode the word plus feedback). Again, all target words were presented in random order. This step was introduced to the child as the “speed reading game.” One block of untimed reading followed by one block of time-limited exposure duration reading was considered a cycle, and this cycle was repeated three times.

#### Post-test measures

After the three learning cycles were completed, two post-tests were conducted to measure orthographic learning using both spelling and orthographic choice tasks. For the spelling task, the tester dictated all trained words in a random order. The children were asked to write down the elves' names exactly as they had learned them on the computer. For the orthographic choice task, each target item (e.g., *ferb*) was presented together with its homophonic foil (e.g., *furb*) and two visual distractors (e.g., *ferq, furq*) on one A4 sheet of paper. The children were asked to choose the correct spelling of the elf's name that they had learned from those four options. These two tasks were measured immediately after the learning trials and again after an hour to increase assessment reliability and statistical power. Thus, eight was the maximum score across two testing points for the orthographic choice task and spelling task for each word type—regular and irregular.

### Results: orthographic learning

#### Learning cycles: untimed and time-limited exposure duration measures

Table 2 summarizes results of the orthographic learning trials for untimed and time-limited exposure duration reading for the two profiles of poor readers and the controls. We aimed to examine the improvement in learning over cycles for regular and irregular items between the three groups of children with different reading profiles. We ran a repeated measures ANOVA with cycle (1, 2, 3), regularity (regular items, irregular items), and exposure duration (untimed, time-limited) as within-subject factors, and group (phonological profile, surface profile, controls) as a between-subject factor. We specified two planned contrasts on the between-subject factor in order to compare the performance of the three different groups. The first contrast compared the performance of the two poor reader groups with the controls and the second contrast compared the performance of the two poor reader groups.

**Table 2:** **Reading accuracy across learning cycles**.

**Reading accuracy**	**Regular**			**Irregular**		
	**Phonological profile**	**Surface profile**	**Controls**	**Phonological profile**	**Surface profile**	**Controls**
**CYCLE 1 (RAW SCORE/4)**						
Untimed	2.78 (1.20)	2.56 (1.13)	3.33 (0.71)	1.22 (0.83)	1.78 (1.20)	2.78 (0.97)
Time-limited	2.67 (1.41)	2.56 (1.13)	3.89 (0.33)	1.89 (0.78)	1.78 (1.56)	3.11 (1.05)
**CYCLE 2 (RAW SCORE/4)**						
Untimed	3.11 (1.17)	3.33 (0.71)	4.00 (0.00)	1.33 (0.87)	2.11 (1.17)	3.56 (0.53)
Time-limited	3.44 (0.73)	2.67 (1.00)	3.89 (0.33)	2.22 (0.44)	2.11 (1.36)	3.22 (0.83)
**CYCLE 3 (RAW SCORE/4)**						
Untimed	3.33 (0.87)	3.33 (0.71)	4.00 (0.00)	2.00 (1.12)	2.11 (1.36)	3.22 (0.97)
Time-limited	3.33 (1.12)	3.22 (0.97)	3.67 (0.50)	2.33 (1.32)	2.11 (1.17)	3.56 (0.53)
**TOTAL (RAW SCORE/12)**						
Untimed	9.33 (3.00)	9.33 (2.24)	11.33 (0.71)	4.56 (2.40)	6.00 (3.35)	9.78 (1.56)
Time-limited	9.44 (3.00)	8.44 (2.60)	11.44 (0.88)	6.44 (2.07)	6.00 (3.71)	10.00 (1.58)

We found a main effect of cycle, *F*_(2, 25)_ = 19.46, *p* < 0.01, η^2^_*p*_ = 0.45, but no interaction between cycle and group, *Fs* < 1; nor were any of the higher level interactions between cycle and group with either regularity or/and exposure duration significant (*Fs* < 1). This indicates that across regular and irregular words, untimed and time-limited exposure conditions, all three groups improved over the learning cycles and that the degree of improvement did not differ between the groups.

The main effect of regularity was significant, *F*_(1, 24)_ = 28.39, *p* < 0.01, η^2^_*p*_ = 0.54, but the interaction between regularity and group was not, *F*_(1, 24)_ = 1.79, *p* = 0.19. All three groups performed better on regular words than on irregular words. However, the interaction between regularity and exposure duration was significant, *F*_(1, 24)_ = 5.48, *p* = 0.03, η^2^_*p*_ = 0.19. Considering the patterns of means across the conditions, this interaction indicated that for regular words, performance did not differ for untimed and time-limited exposure duration, *t*_(1, 26)_ = 0.77, *p* = 0.45. However, for irregular words, performance was be better under the time-limited condition than under the untimed exposure duration, *t*_(1, 26)_ = −2.23, *p* = 0.03. There was also an interaction between exposure duration and group, *F*_(2, 24)_ = 4.33, *p* = 0.03. The interaction reflected the fact that for the control and surface group, there were no differences between exposure duration [control: *t*_(1, 8)_ = 0.54, *p* = 0.61; surface: *t*_(1, 8)_ = 1.04, *p* = 0.33]; but for the phonological group performance was better in the time-limited condition compared to the untimed condition, *t*_(1, 8)_ = 3.46, *p* < 0.01.

Finally, there was a main effect of group, *F*_(2, 24)_ = 8.56, *p* < 0.01, η^2^_*p*_ = 0.42. The first planned contrast (both poor reader groups vs. controls) showed that, across conditions (regular/irregular, untimed/time-limited), the controls performed better than the poor reader groups, *F*_(1, 24)_ = 17.12, *p* < 0.01, η^2^_*p*_ = 0.42. However, there was no difference in overall performance between the two poor reader groups, *Fs* < 1.

It should be noted that the performance of the control group is at ceiling on the regular items at the later cycles and hence did not meet the statistical assumption of equal variance. Therefore, we ran a nonparametric randomization test that does not make any assumptions about the distribution of the data (Lunneborg, 2001). This randomization test was conducted for the regular as well as the irregular items on the main effect of group. The results confirmed that across cycle and exposure duration conditions, the controls performed better than the two poor reader groups (regular: *p* = 0.03; irregular: *p* < 0.01).

In summary, it was found that all three groups improved over learning cycles, but across learning cycles, the controls performed better than both poor reader groups. Importantly, the performance of the children with phonological and surface profiles did not differ. In addition, all three groups performed better on items with regular pronunciations than those with irregular pronunciations, and there was no difference in this regularity effect between the surface and phonological profiles. However, we need to interpret the results with caution as the controls were at ceiling for the regular items in the untimed reading condition. Lastly, it was found that for irregular words but not regular words, the performance was better under the time-limited exposure duration condition than under the untimed condition, particularly for the phonological group. This can be explained by the fact that the untimed condition provides an opportunity to decode a word, and in the case of irregular items, decoding results in incorrect responses. This result indicated that the timed condition has minimal influence from phonological decoding.

#### After learning cycles: spelling and orthographic choice measures

Table 3 summarizes results of the spelling and orthographic choice measures. We ran repeated measures ANOVAs with word regularity (regular items, irregular items) as a within-subject factor and group (phonological profile, surface profile, controls) as a between-subject factor. We specified the same two planned contrasts on the between-subject factor to compare the performance of the three different groups. Analyses were conducted separately for the spelling and orthographic choice tasks.

**Table 3:** **Accuracy on Spelling and Orthographic Choice Measured after the Learning Cycles (with SDs in brackets)**.

	**Phonological profile**	**Surface profile**	**Controls**
**REGULAR ITEMS (RAW SCORE/8)**			
Spelling	6.67 (1.58)	5.33 (2.00)	7.33 (1.12)
Orthographic choice	7.67 (0.50)	5.44 (1.94)	7.67 (0.71)
**IRREGULAR ITEMS (RAW SCORE/8)**			
Spelling	6.44 (0.88)	4.44 (2.19)	6.33 (1.12)
Orthographic choice	7.11 (1.05)	4.89 (2.15)	7.22 (0.97)

For the spelling task, the main effect of regularity approached significance, *F*_(1, 24)_ = 4.20, *p* = 0.052, η^2^_*p*_ = 0.15, and there was no interaction between regularity and group (*F*s < 1). The main effect for group was significant, *F*_(2, 24)_ = 6.11, *p* < 0.01, η^2^_*p*_ = 0.34. The planned contrasts showed that this group main effect was reflecting the significantly lower performance of the surface group compared to the controls, *F*_(1, 24)_ = 10.44, *p* < 0.01, η^2^_*p*_ = 0.30, as well as the phonological group, *F*_(1, 24)_ = 7.67, *p* = 0.01, η^2^_*p*_ = 0.24. The phonological group on the other hand, performed equally as well as the controls, *Fs* < 1.

For the orthographic choice task, the difference between regular and irregular items was not significant, *F*_(1, 24)_ = 2.61, *p* = 0.12, nor was the interaction between word regularity and group, *Fs* < 1. As with spelling performance, there was a main effect of Group, *F*_(2, 24)_ = 12.97, *p* < 0.01, η^2^_*p*_ = 0.52. The planned contrasts showed that the surface group was worse on the orthographic choice task compared to the controls, *F*_(1, 24)_ = 19.93, *p* < 0.01, η^2^_*p*_ = 0.45, and compared to the phonological group, *F*_(1, 24)_ = 18.97, *p* < 0.01, η^2^_*p*_ = 0.44. Again, the phonological group performed at the same level as the controls, *Fs* < 1. An additional analysis confirmed that all three groups performed above chance level (25% accuracy), even the surface group, *t*_(1, 8)_ = 7.54, *p* < 0.01.

In summary, the pattern of results of the spelling task was consistent with that of the orthographic choice task. For both tasks, the phonological group did not perform differently from the controls whereas the surface group was worse than both the phonological group and the control group. However, in contrast to the findings of the learning trials, the children did not perform better on the regular items than on the irregular items for the orthographic choice task and the difference in performance only approached significance for the spelling task.

### Discussion

The first question of interest in Study 1 was whether children with reading difficulties are able to learn novel words at all or to learn at the same pace as typically developing readers. We found that, just like the controls, children with both types of reading profile showed learning over the learning cycles as evidenced by untimed and time-limited reading accuracy, and the rate of improvement was no different from controls. This is in contrast with previous studies that have found little or no evidence of orthographic learning in children with dyslexia (Reitsma, 1983, 1989; but also see Staels and van den Broeck, 2013). The difference between the results of the present and previous studies may be explained by the sensitivity of the measure of orthographic learning. In the current study orthographic learning was monitored online during the exposure trials, whereas Reitsma (1983, 1989) found that Dutch poor readers showed no evidence of orthographic learning measured by an orthographic choice task after learning took place. However, we found that although the two dyslexic groups showed evidence of orthographic leaning, their reading accuracy overall was worse than the controls, and this is in line with previous studies using a group of mixed dyslexics (e.g., Manis, 1985; Share and Shalev, 2004).

The second question of interest was to explore the contrasting predictions made by the hypothesis of phonological decoding as the primary factor in orthographic learning vs. that of the children's reading profiles. According to the phonological decoding hypothesis, the phonological group, with their impaired phonological decoding skill, would be predicted to perform less well than the controls and the surface group. In contrast, the subtype profiles of the two groups predict that the phonological group would be better at orthographic learning than the surface group based on their superior sight word reading ability. The results of the present study did not fully support either of the hypotheses but was more consistent with the children's subtype profiles. The performance of the two poor reader groups did not differ on either untimed or time-limited exposure duration reading accuracy. However, the phonological group was found to be no different from the control group and to outperform the surface group on the subsequent spelling and orthographic choice task. The surface group was also significantly worse than the controls on the spelling and orthographic choice tasks. It should be noted that both the control and the phonological group performed close to ceiling on the orthographic choice tasks, hence making the differences between the two groups hard to detect. Nevertheless, the orthographic learning results of these two groups were in line with the selective difficulties in their reading profiles within the framework of the dual route model. The results of Study 1 thus showed that the difference in phonological decoding skill between the two subtype profiles did not directly translate into differences in the ability to acquire novel word representations, which is in contrast with the hypothesis that phonological decoding is the primary determinant of successful orthographic learning.

It is important to note that the surface group did not outperform the phonological group on untimed reading accuracy and did perform worse than the control group. We did not expect this result as we preselected the surface group to have average phonological decoding skills. The finding that the two poor reader groups did not differ in untimed reading accuracy might be explained by the test items that were used. For this task we used only four, regular, four to five letter nonwords. It might be the case that the test was not sensitive enough and/or might not have had enough statistical power to differentiate between the two poor reader groups. In addition, the finding that the surface group was worse at decoding the novel words than the control group could be due to the superior decoding ability of the control group. That is, although the surface group was within the average range on the nonword reading selection measure (*z* = −0.57, *SD* = 0.21), they were still on average worse than controls (*z* = 0.15, *SD* = 0.66), *t*_(1, 17)_ = −2.47, *p* = 0.03.

The last aim of Study 1 was to examine the effect of word regularity on orthographic learning. All groups showed higher reading accuracy when learning regular items than when learning irregular items. This implies that phonological decoding *was* used by both poor reader groups, as well as the typical readers, during the orthographic learning process. Together these results suggest that phonological decoding plays a role in orthographic learning for both subtype groups, yet it is also clear that this skill is not sufficient to fully account for the success of orthographic learning.

If phonological decoding skill cannot fully explain successful orthographic learning then what are the other factors determining this learning process? In order to further explore orthographic learning in poor readers, the second half of this study investigated the predictors of orthographic learning beyond phonological decoding. It has been proposed that poor readers could be relying on alternative learning strategies in order to compensate for poor phonological decoding skills (Stanovich and Siegel, 1994; Siegel et al., 1995; Castles et al., 1999). For example, it is possible that for children who have difficulties with phonological decoding, vocabulary knowledge is relied on more heavily during orthographic learning. In support of this, previous studies have found that when decoding can only be partially successful, in the case of irregular novel words, contextual information and vocabulary knowledge play a role in orthographic learning (Wang et al., 2011, 2013; Duff and Hulme, 2012). Similarly, word meaning has also been found to assist the reading of irregular words (e.g., Nation and Snowling, 1998; Ouellette, 2006; Bowey and Rutherford, 2007; Ricketts et al., 2007; McKay et al., 2008; Nation and Cocksey, 2009).

In addition to phonological decoding skills and vocabulary knowledge, pre-existing orthographic knowledge has also been considered an important factor in orthographic learning (Cunningham et al., 2002; Conners et al., 2010). Share (1995) suggested that although phonological decoding is the primary component of orthographic learning, the secondary, orthographic component determines how quickly and accurately orthographic representations are acquired.

In sum, we aimed to develop a more detailed picture of the reading processes associated with orthographic learning by exploring the strengths of the relationship between different reading and language skills and orthographic learning of regular and irregular words.

## Study 2

For the purpose of exploring how well different skills involved in reading predict orthographic learning of regular and irregular words within a larger group of poor readers, we again drew on the language and reading skills in the dual route model as we did for subgroup validation in Study 1 (see Figure 1 earlier).

As noted earlier, phonological decoding is often assumed to be the key to orthographic learning. Based on this hypothesis, we predicted that skills reflecting nonlexical processing (phonological decoding in particular) would be important for orthographic learning of both regular words and irregular words. However, when accurate decoding is compromised or not possible (such as when phonological decoding skill is impaired or when words are irregular), knowledge of semantics, phonology or orthography may become more important.

### Methods

#### Participants

The same cohort of 91 poor readers screened in Study 1 participated in Study 2. As mentioned in Study 1, these children scored at least one standard deviation below average for their age on one or more of the two subscales (irregular word and nonword reading) of the Castles and Coltheart 2 word reading test (CC2; Castles et al., 2010a). On average, the children scored −1.59 (*SD* = 0.65) on nonword reading; and −1.40 (*SD* = 0.67) on irregular word reading.

#### Materials and procedure

We assessed the poor readers on tasks tapping the six basic components of the dual-route model. In addition, all children completed the same orthographic learning task described in Study 1. In order to increase statistical power for the analyses used in Study 2, we created another set of nonword stimuli, consisting of an additional four regular and four irregular items. The extra set of items was created in the same way as described in Study 1. The same procedure of the orthographic learning task was applied in a separate session 8 weeks after the first set of nonwords were learnt. Each child was tested individually in a quiet room, and the children took approximately 100–120 min to complete all the assessments. The results of the six reading and language skill measures were used as predictors, and the orthographic learning performances were used as outcome measures.

### Results

To investigate how well each reading subcomponent predicts orthographic learning, a set of correlations, followed by stepwise multiple regressions, was conducted with the dual route processing components as predictors, and the various orthographic learning measures as the dependent variables. Regressions were carried out in addition to correlations as the predictor tasks are themselves intercorrelated, in order to identify the relationship between these factors and orthographic learning outcomes when the intercorrelations between the variables are controlled.

Table 4 shows the results of a series of correlations and partial correlations controlling for age and non-verbal IQ between the outcomes of the orthographic learning task (outcome measures: no. 1–8) and the components involved in lexical, nonlexical, and both routes (predictors: no. 9–14). Before the effects of age and non-verbal IQ were partialled out, all of the components involved in the reading routes correlated with almost all measures of orthographic learning. After controlling for age and IQ, the main difference is that the associations between orthographic learning measures and semantic knowledge (PPVT), and phonological lexicon functioning (ACE) were no longer significant.

**Table 4:** **Correlations of the outcome measures and the predictors with (below the diagonal line) and without (above the diagonal line) age and nonverbal IQ controlled**.

		**1**	**2**	**3**	**4**	**5**	**6**	**7**	**8**	**9**	**10**	**11**	**12**	**13**	**14**	**15**	**16**
Regular	1. Untimed reading	–	**0.93**	**0.68**	**0.44**	**0.60**	**0.69**	**0.63**	**0.55**	**0.45**	**0.60**	**0.61**	**0.41**	**0.41**	**0.52**	**0.53**	**0.43**
	2. Time-limited reading	**0.89**	–	**0.71**	**0.52**	**0.56**	**0.69**	**0.65**	**0.59**	**0.46**	**0.55**	**0.64**	**0.40**	**0.43**	**0.48**	**0.52**	**0.40**
	3. Spelling	**0.57**	**0.65**	–	**0.60**	**0.42**	**0.61**	**0.71**	**0.62**	**0.48**	**0.47**	**0.65**	**0.35**	**0.32**	**0.38**	**0.45**	**0.45**
	4. Orthographic choice	**0.38**	**0.48**	**0.62**	–	**0.47**	**0.56**	**0.56**	**0.62**	**0.46**	**0.40**	**0.46**	0.19	**0.29**	**0.39**	0.17	**0.28**
Irregular	5. Untimed reading	**0.53**	**0.47**	**0.33**	**0.45**	–	**0.85**	**0.63**	**0.56**	**0.32**	**0.45**	**0.49**	**0.29**	**0.31**	**0.34**	**0.36**	0.18
	6. Time-limited reading	**0.58**	**0.58**	**0.53**	**0.53**	**0.83**	–	**0.73**	**0.65**	**0.50**	**0.47**	**0.58**	**0.36**	**0.36**	**0.41**	**0.44**	**0.28**
	7. Spelling	**0.48**	**0.53**	**0.59**	**0.52**	**0.60**	**0.69**	–	**0.73**	**0.53**	**0.53**	**0.62**	**0.37**	**0.31**	**0.32**	**0.40**	**0.46**
	8. Orthographic choice	**0.38**	**0.44**	**0.53**	**0.61**	**0.50**	**0.56**	**0.68**	–	**0.51**	**0.46**	**0.63**	**0.41**	**0.32**	**0.30**	**0.41**	**0.39**
Predictors	9. Letter analysis	**0.25**	**0.27**	**0.36**	**0.38**	0.21	**0.38**	**0.39**	**0.37**	–	**0.36**	**0.44**	**0.27**	**0.27**	**0.31**	**0.39**	**0.44**
	10. Letter-sound know.	**0.45**	**0.38**	**0.33**	**0.32**	**0.36**	**0.33**	**0.39**	**0.34**	0.19	–	**0.41**	**0.38**	**0.38**	**0.32**	**0.39**	**0.33**
	11. Orthog. lexicon	**0.41**	**0.47**	**0.52**	**0.44**	**0.35**	**0.43**	**0.49**	**0.51**	**0.27**	**0.22**	–	**0.52**	**0.49**	**0.41**	**0.55**	**0.38**
	12. Semantics	0.09	0.08	0.05	0.07	0.09	0.11	0.11	0.14	−0.01	0.20	**0.26**	–	**0.67**	**0.30**	**0.22**	**0.48**
	13. Phono. lexicon	0.09	0.11	0.07	0.17	0.14	0.13	0.03	0.06	−0.01	0.16	**0.24**	**0.50**	–	**0.31**	**0.49**	**0.44**
	14. Phonemic buffer	**0.51**	**0.44**	**0.39**	**0.35**	**0.30**	**0.36**	**0.28**	**0.24**	**0.25**	**0.26**	**0.39**	**0.25**	**0.23**	–	0.16	0.13
	15. Age	–	–	–	–	–	–	–	–	–	–	–	–	–	–	–	**0.48**
	16. Non-verbal IQ	–	–	–	–	–	–	–	–	–	–	–	–	–	–	–	–

*Predictors 9–14 refer to the six reading components tested by: Cross-case copying (Letter Analysis, 9); Letter-sound test (Letter-sound knowledge; 10); Door/Doar Lexical Decision (Orthographic lexicon, 11); PPVT (Semantic knowledge, 12); ACE (Phonological lexicon, 13); NEPSY (Phonemic buffer, 14); Age (15); Non-verbal IQ (K-Bit, 16)*.

*Values represent Pearson's correlation coefficients; numbers in bold indicate p < 0.05, 2-tailed*.

The results of the regression analyses are summarized in Table 5. In the first step, age and non-verbal IQ were entered, followed by all the other potential predictor variables at step 2. Overall, letter-sound knowledge and orthographic knowledge seemed to be the best predictors of orthographic learning. Untimed exposure duration reading accuracy was predicted by letter-sound knowledge and phonemic buffer efficiency. Time-limited exposure duration reading accuracy was predicted by letter-sound knowledge, phonemic buffer efficiency, and word-specific orthographic knowledge (functioning of orthographic lexicon). Spelling accuracy was predicted by letter-sound knowledge and orthographic knowledge, whereas orthographic choice accuracy was only predicted by orthographic knowledge. For irregular items, orthographic knowledge was the only significant predictor for all measures except untimed exposure duration reading accuracy, which was predicted by both orthographic knowledge and letter-sound knowledge.

**Table 5:** **Summary of regression results predicting orthographic learning of regular and irregular words from lexical and nonlexical processing components**.

	**Step**	***R*^2^**	**Beta**							
			**Age**	**Nonverbal IQ**	**Letter analysis**	**Letter-sound knowledge**	**Orthogr-aphic knowledge**	**Semantic knowledge**	**Phonological knowledge**	**Phonological buffer**
**REGULAR ITEMS**										
Untimed reading	1	0.22	0.34^*^	0.23	0.17	0.43^*^	0.18	−0.19	0.14	0.34^*^
	2	0.66	0.20	0.04						
Time-limited reading	1	0.19	0.35^*^	2.57	0.11	0.34^*^	0.32^*^	−0.25	0.06	0.28^*^
	2	0.54	0.24	0.04						
Spelling	1	0.22	0.15	0.40^*^	0.05	0.27^*^	0.54^*^	−0.13	−0.10	0.17
	2	0.57	−0.07	0.29^*^						
Orthographic choice	1	0.15	0.05	0.37^*^	0.01	0.15	0.52^*^	−0.06	−0.07	0.15
	2	0.43	−0.16	0.28^*^						
**IRREGULAR ITEMS**										
Untimed reading	1	0.13	0.30^*^	0.12	0.21	0.29^*^	0.40^*^	−0.23	0.10	0.14
	2	0.46	0.15	0.00						
Time-limited reading	1	0.17	0.33^*^	0.16	0.11	0.18	0.37^*^	−0.12	0.20	0.21
	2	0.51	0.15	0.02						
Spelling	1	0.28	0.19	0.44^*^	0.10	0.20	0.50^*^	−0.04	0.00	0.07
	2	0.57	−0.06	0.31^*^						
Orthographic choice	1	0.31	0.25	0.42^*^	0.09	0.08	0.56^*^	0.05	0.01	−0.05
	2	0.59	−0.04	0.32^*^						

* *p < 0.05*.

It should be noted that although it seemed that letter-sound knowledge was a better predictor of performance for the regular items than the irregular items, and that orthographic knowledge was a better predictor for irregular items than regular items, these correlational differences between regular and irregular items did not reach significance. However, across the two predictors, orthographic knowledge was a significantly better predictor than letter-sound knowledge for scores on spelling and orthographic choice measures, regardless of word regularity (regular spelling: *z* = −2.17, *p* = 0.03; irregular spelling: *z* = −2.30, *p* = 0.02; regular orthographic choice: *z* = −2.82, *p* < 0.01; irregular orthographic choice: *z* = −3.67, *p* < 0.01).

### Discussion

The results showed that letter-sound knowledge predicted the outcomes of all measures assessing regular word learning except for orthographic choice. Letter-sound knowledge also predicted the untimed reading accuracy during irregular word learning, but it did not predict any other measures assessing irregular word learning. The ability to repeat nonwords was used as an index of phonemic buffer proficiency, and performance on this task predicted orthographic learning of regular words when learning was measured by accuracy in reading aloud (both timed and untimed).

Our measure of orthographic knowledge predicted success on our dynamic measures of orthographic learning of both regular and irregular words except for untimed reading accuracy of regular words. The fact that orthographic knowledge predicted time-limited but not untimed accuracy in reading the regular items is interesting as it suggests that, when rapid and fluent access to the orthographic representation is required, orthographic knowledge may play a more important role than when reading is untimed. In addition, orthographic knowledge was a better predictor than letter-sound knowledge when orthographic learning was measured by spelling and orthographic choice tasks. Finally, in contrast to the prediction that poor readers may use alternative skills such as vocabulary knowledge when learning to read, better functioning of the semantic system and/or phonological lexicon did not predict better orthographic learning.

## General discussion

In this paper we examined orthographic learning in poor readers. Study 1 focused on orthographic learning of regular and irregular novel words in children with surface and phonological reading profiles. We developed a novel paradigm to track orthographic learning online. Participants were first asked to read the presented novel words untimed, then the items were presented under time-limited exposure duration of 200 ms. This cycle was repeated three times and followed by spelling and orthographic choice tasks. This set up allowed us to track orthographic learning more dynamically (i.e., untimed and time-limited reading accuracy) than traditional measures (such as spelling and orthographic choice), that typically take place after learning has taken place. With our novel and traditional measures of orthographic learning, we aimed to examine the role of phonological decoding in orthographic learning.

More specifically, we wanted to investigate whether phonological decoding is primary in orthographic learning as is widely proposed (e.g., Brady and Shankweiler, 1991; Byrne, 1992, 1998; Share, 1995). In this context, the orthographic learning of two subgroups is particularly interesting: children with specific difficulties with phonological decoding (a phonological profile) and those with specific difficulties in orthographic knowledge (a surface profile). If phonological decoding is indeed primary to orthographic learning, this should result in poorer orthographic learning in children with a phonological profile and normal orthographic learning in children with a surface profile. In addition to our measures of orthographic learning, we also examined the degree to which the different poor reader groups relied on phonological decoding by comparing the difference in their performance on regular and irregular word learning.

We found that children with phonological and surface profiles showed the same amount of orthographic learning on the dynamic measures (scores on untimed and time-limited trials). However, orthographic learning was still less efficient overall compared to that of the age-matched controls. This finding is consistent with previous studies suggesting that poor readers take longer to learn to read novel words (e.g., Manis, 1985; Share and Shalev, 2004). The results of our study add evidence that this less efficient orthographic learning is already apparent during online learning trials. The finding that children with a surface profile have superior phonological decoding ability but did not outperform children with a phonological profile seems to be inconsistent with the view of phonological decoding as the primary factor for orthographic learning. However, a key feature of the self-teaching hypothesis can also explain this finding. According to this hypothesis, orthographic learning is item based. Consequently, what is relevant to the success of orthographic learning is the correct decoding of the items to be learnt rather than one's phonological decoding ability in general. On the untimed trials in our dynamic measure, the children with a surface profile did not decode the words better than the children with a phonological profile. In other words: they did not show superior decoding skills on this task to start with. Hence, in this regard it is not surprising that they did not do better than the children with phonological dyslexia on the time-limited exposure duration trials.

Our findings on the traditional measures (spelling and orthographic choice) painted a different picture. Here the children with a surface profile performed more poorly than both the children with a phonological profile and the controls, which is consistent with what was found by Castles and Holmes (1996). This result is also consistent with the prediction based on the two groups' reading difficulties within the framework of the dual route model: the phonological group had normal sight word reading ability and the surface group had impaired sight word reading ability. Moreover, the children with a phonological profile performed as well as the controls despite their poorer performance on reading accuracy during the learning trials. This imbalance between decoding performance and orthographic learning results suggests again that orthographic learning ability cannot be explained by phonological decoding ability alone.

One possible explanation for the inconsistent performance of the phonological group across dynamic and traditional measures is that different task demands are imposed by the different orthographic learning tasks used for this study. More specifically, the dynamic measures (untimed and time-limited reading aloud) required verbal output whereas the traditional measures (spelling and orthographic choice) did not. As phonological impairment in reading is often associated with deficit in verbal output (Hulme and Snowling, 1992; Szenkovits and Ramus, 2005), it is not surprising that the phonological group performed worse than typical readers on measures requiring verbal output compared to those not requiring verbal output. This explanation is also consistent with findings from Study 2 (results will be discussed in more detail later), where performance on dynamic measures was more strongly associated with letter-sound knowledge and phonemic buffer functioning than was performance on traditional measures.

The role of phonological decoding in orthographic learning was also examined by manipulating word regularity. We found word regularity effects for the dynamic measures (untimed and time-limited reading) but not for the traditional post-test measures (spelling and orthographic choice). Moreover, these effects were the same for all groups. The regularity effect found in dynamic measures suggests that phonological decoding does play a role during the learning process, even for children with a phonological profile. However, the word regularity effect was not significant for any of the post-test measures. The absence of a regularity effect for spelling and orthographic choice is in line with outcomes of studies examining regularity effects in word recognition. These studies showed that regularity effects are restricted to tasks involving reading aloud and typically not found in word recognition tasks, such as lexical decision (e.g., Coltheart et al., 1979; Seidenberg et al., 1984; Schmalz et al., 2013; but see Parkin, 1982).

Study 2 explored to what degree the different skills that underlie reading predicted orthographic learning of regular and irregular words in poor readers. We selected the underlying component skills from the dual-route model of reading as predictors, and as outcome variables we used the dynamic and traditional orthographic learning measures. According to the view that phonological decoding is the primary factor to successful orthographic learning, we expected that skills reflecting nonlexical processing (letter-sound knowledge in particular) would be stronger predictors of orthographic learning of both regular and irregular words than skills reflecting lexical processing. We found that letter-sound knowledge is indeed a strong predictor of orthographic learning.

However, we did not find letter-sound knowledge to be a stronger predictor of orthographic learning than skills reflecting lexical processing. In fact, we found that orthographic knowledge, a lexical processing factor, was a good predictor of both regular and irregular word learning, and an even better predictor than letter-sound knowledge for spelling and orthographic choice. The association between orthographic knowledge and orthographic learning appeared to be particularly evident when the measure of learning directly tapped word-specific (spelling and orthographic choice) and fluent (timed-limited reading) access of orthographic representations.

Thus, both Study 1 and 2 showed that orthographic knowledge was associated with success in orthographic learning. In Study 1, we found that children with average phonological decoding skill but good orthographic knowledge showed normal orthographic learning on the traditional learning measures. In contrast, children with an opposite reading profile—impaired orthographic knowledge but good phonological decoding skill showed impaired orthographic learning. In Study 2, we found that orthographic knowledge significantly predicted orthographic learning even after phonological decoding skills were controlled for. Orthographic knowledge also appeared to be a stronger predictor than phonological decoding skill when orthographic learning was measured by traditional measures (spelling and orthographic choice). Together these findings suggest that orthographic knowledge is not only important in orthographic learning, but also that having impaired orthographic knowledge could be more detrimental than having impaired phonological decoding skill when learning new words. The importance of orthographic knowledge in orthographic learning has also been reported in previous studies with typically developing readers (Cunningham, 2006; Conners et al., 2010). Similarly, the self-teaching hypothesis suggests that, although phonological decoding provides the opportunity for orthographic learning to take place, orthographic knowledge is the secondary factor required for successful orthographic learning (Share, 1995, 2011). Our results support the view that orthographic knowledge is important in orthographic learning, but challenge the view that orthographic knowledge is a “secondary” factor. It seems that orthographic knowledge may actually be equally important or even more important than phonological decoding in building up orthographic representations.

However, it must be considered in this context that there are two alternative explanations as to why orthographic knowledge might be a significant predictor of orthographic learning. First, the way orthographic knowledge was measured in this study could be seen as a measure of the children's historic ability to acquire orthographic representations. Hence, the relationship between orthographic knowledge and orthographic learning could simply be that both are a reflection of the children's ability to acquire lexical representations: children with better abilities to acquire orthographic representations will be better at both the task tapping the orthographic lexicon (as a result of past orthographic learning) and at our orthographic learning task (current orthographic learning). Second, it could be that existing orthographic representations contribute to the actual process of acquiring new representations with children using this knowledge during the learning process. This might occur by using analogies of known words or utilizing familiarity of orthographic patterns. Further research is required to investigate how exactly existing orthographic knowledge assists orthographic learning of novel words.

As mentioned earlier, one might expect that for poor readers, the success of orthographic learning might rely on alternative skills, such as semantics, compensating for poor phonological decoding skills. In contrast to this prediction, this study did not find any evidence that pre-existing semantic and phonological knowledge (measured by PPVT-IV and ACE6-11) predicted orthographic learning of regular or irregular words. However, the orthographic learning paradigm in this study did not provide word-specific vocabulary (semantic and phonological) knowledge for the novel words. Hence, there was little opportunity for the children to use such skills. It would be interesting for future studies to explore whether word-specific vocabulary knowledge affects orthographic learning in poor readers. Further investigation using a design that provides vocabulary knowledge of the novel words prior to written exposure, such as that used in Wang et al. (2011, 2013), is needed.

There are a number of limitations of the present study that require further consideration. First, due to the manipulation of word regularity, the items were read to the children as an initial exposure before the learning cycles started, and during the learning cycles feedback was provided. Consequently, although after the initial exposure the children were asked to first read the target words by themselves to simulate a partial self-teaching paradigm, it was not an independent learning environment. Therefore, it is possible that the results would have been different had the children learned the words without the experimenter's input. For example, the children with a phonological dyslexia profile may have benefited from the input and feedback to compensate for their poor decoding of the regular items, resulting in their untimed and time-limited reading accuracy not being different from the children with a surface dyslexia profile. Although providing feedback is still ecologically valid, as children often receive feedback when learning to read, particularly with irregular words, we cannot interpret the results in the context of pure self-teaching.

Second, it is possible that factors beyond phonological decoding and orthographic knowledge influenced the pattern of impaired orthographic learning in surface dyslexia and normal orthographic learning in phonological dyslexia. According to the self-teaching hypothesis (Share, 1995), phonological decoding draws the reader's attention to the order and identity of the letters in the word, and produces the phonology. This then allows bonding to occur between the phonology and the orthography via some kind of associative learning procedure. It is possible that differences in the ability to establish associations between phonology and orthography are the source of the difference in orthographic learning skill between the two subtypes of dyslexia. In studies that did not make distinctions between subtypes, children with dyslexia were found to have difficulties in learning paired associations (Gascon and Goodglass, 1970; Vellutino et al., 1975; Messbauer and de Jong, 2003; Litt et al., 2013). In addition, other abilities may also contribute to individual differences in learning to read, such as mapping the output of letter-sound correspondences to existing phonology of a word (e.g., *was:* from/w..aa..ss/… to /woz/; Elbro et al., 2012), and capitalizing contextual and syntactic information (Tunmer et al., 1987; Tunmer and Chapman, 2004). Future studies are required to further investigate orthographic learning in children with subtypes of reading profiles.

In sum, Study 1 used a novel paradigm that allowed us to explore the role of phonological decoding and track orthographic learning in two groups of poor readers who had contrasting reading impairments. The two poor reader groups showed orthographic learning patterns that were consistent with their reading profiles, which suggested that phonological decoding skill alone is insufficient for acquiring orthographic representations. Study 2 was the first to break down components of reading processes based on the dual route model of reading and to use these components to explore factors associated with orthographic learning. The results of this study indicated that, in addition to phonological decoding (letter-sound knowledge), prior orthographic knowledge also predicted the success of orthographic learning. Together, the outcomes of the two studies suggest that phonological decoding plays a role in orthographic learning of both regular and irregular words, and for children with and without phonological decoding difficulties. Orthographic knowledge was also found to be important in orthographic learning, especially when the measures of learning directly tapped word-specific and fluent access to orthographic representations.

### Conflict of interest statement

The authors declare that the research was conducted in the absence of any commercial or financial relationships that could be construed as a potential conflict of interest.

## Footnotes

^1^Bailey et al. (2004) also used a spelling task to measure orthographic learning, however, due to the fact that accuracy of spelling was at floor, only reading accuracy results were discussed.
