# Supplementary material for: Tracking orthographic learning in children with different profiles of reading difficulty
Source: Front Hum Neurosci. 2014 Jul 4;8:468. doi: 10.3389/fnhum.2014.00468 (PMC4081833; doi:10.3389/fnhum.2014.00468)
Supplement: Supplementary file 1 [file DataSheet1.DOCX]

| Target items and the foils for the orthographic choice task for Study 1 & 2 | | | | | | |
| --- | --- | --- | --- | --- | --- | --- |
|  | Word type | Target items | | Phonologically- related foils | Visual Distractor 1 | Visual Distractor 2 |
| Set 1 | Regular | ferb | (/fɜ:b/) | furb | ferq | furq |
|  | Regular | crade | (/kreɪd/) | craid | crafe | craif |
|  | Regular | smope | (/sməʊp /) | smoap | snope | snoap |
|  | Regular | dowd | (/daʊd/) | doud | douf | dowf |
|  | Irregular | broon | (/brʌn/) | brunn | broop | brupp |
|  | Irregular | fape | (/fæp/) | fapp | fabe | fabb |
|  | Irregular | cleap | (/kleɪp/) | clape | cleag | clage |
|  | Irregular | vack | (/va:k /) | vark | vock | vork |
| Set 2 | Regular | kerg | (/kɜ:g/) | kurg | kerp | kurp |
|  | Regular | plabe | (/pleɪb/) | plaib | plafe | plaif |
|  | Regular | spobe | (/spəʊb /) | spoab | spode | spoad |
|  | Regular | lowk | (/laʊk/) | louk | lown | loun |
|  | Irregular | smoof | (/smʌf/) | smuff | swoof | swuff |
|  | Irregular | mafe | (/mæf/) | maff | mage | magg |
|  | Irregular | greaf | (/greɪf/) | grafe | greak | grake |
|  | Irregular | gatt | (/ga:t /) | gart | gakk | gark |

Appendix
